# Choose Your Weaponry: Selective Storage of a Single Toxic Compound, Latrunculin A, by Closely Related Nudibranch Molluscs

**DOI:** 10.1371/journal.pone.0145134

**Published:** 2016-01-20

**Authors:** Karen L. Cheney, Andrew White, I. Wayan Mudianta, Anne E. Winters, Michelle Quezada, Robert J. Capon, Ernesto Mollo, Mary J. Garson

**Affiliations:** 1 School of Biological Sciences, The University of Queensland, Brisbane QLD 4072, Australia; 2 School of Chemistry and Molecular Biosciences, The University of Queensland, Brisbane QLD 4072, Australia; 3 Institute for Molecular Bioscience, The University of Queensland, Brisbane QLD 4072, Australia; 4 Istituto di Chimica Biomolecolare, Consiglio Nazionale delle Ricerche, Pozzuoli, Italy; University of New South Wales, AUSTRALIA

## Abstract

Natural products play an invaluable role as a starting point in the drug discovery process, and plants and animals use many interesting biologically active natural products as a chemical defense mechanism against predators. Among marine organisms, many nudibranch gastropods are known to derive defensive metabolites from the sponges they eat. Here we investigated the putative sequestration of the toxic compound latrunculin A—a 16-membered macrolide that prevents actin polymerization within cellular processes—which has been identified from sponge sources, by five closely related nudibranch molluscs of the genus *Chromodoris*. Only latrunculin A was present in the rim of the mantle of these species, where storage reservoirs containing secondary metabolites are located, whilst a variety of secondary metabolites were found in their viscera. The species studied thus selectively accumulate latrunculin A in the part of the mantle that is more exposed to potential predators. This study also demonstrates that latrunculin-containing sponges are not their sole food source. Latrunculin A was found to be several times more potent than other compounds present in these species of nudibranchs when tested by *in vitro* and *in vivo* toxicity assays. Anti-feedant assays also indicated that latrunculin A was unpalatable to rock pool shrimps, in a dose-dependent manner. These findings led us to propose that this group of nudibranchs has evolved means both to protect themselves from the toxicity of latrunculin A, and to accumulate this compound in the mantle rim for defensive purposes. The precise mechanism by which the nudibranchs sequester such a potent compound from sponges without disrupting their own key physiological processes is unclear, but this work paves the way for future studies in this direction. Finally, the possible occurrence of both visual and chemosensory Müllerian mimicry in the studied species is discussed.

## Introduction

Many natural products used in drug discovery are molecules that are used to protect plants and animals from consumers, and investigations into their chemical structure and bioactivity are numerous [1, 2]. Marine organisms, in particular molluscs, are an important source of diverse natural products, and the study of their chemical properties has led to the discovery of many biologically potent chemicals with analgesic, anti-inflammatory, antiviral and anticancer activity. For example, the antitumour depsipeptide kahalalide F was isolated from the opisthobranch mollusc *Elysia rufescens*, and is used by both the mollusc and its dietary alga *Bryopsis* spp. (the true source of kahalalide F) as a chemical defense from predation [3]. Kahalalide F induces cell death via oncosis, and in phase I and II clinical trials has been shown to benefit patients with advanced cancerous tumors [4, 5].

Bioactive compounds also occur in many species of nudibranch mollusc (Mollusca: Gastropoda: Nudibranchia). Among these compounds, latrunculin A, a 16-membered macrolide with an attached 2-thiazolidinone unit, which was originally isolated from the Red Sea sponge *Negombata magnifica* (= *Latrunculia magnifica*) [6], and other Pacific sponges: *Cacospongia mycofijiensis* (*= Spongia mycofijiensis*, *= Petrosaspongia mycofijiensis* [7–9]), and *Hyattela* sp. [10], has also been found in a group of sponge-feeding nudibranchs from the family Chromodorididae, including: *Chromodoris elisabethina* [11], *Chromodoris lochi* [7, 10], *C*. *hamiltoni* [12], and *C*. *quadricolor* [13]. These nudibranchs have been observed apparently feeding on these sponges [14] and acanthorhab sponge spicules, typically found in latrunculid sponges, were found in the gut contents of *C*. *hamiltoni* [15], suggesting that the uptake of latrunculin A is derived from their diet. Latrunculin A exhibits interesting and potent biological properties, e.g. [16, 17]. In particular, this compound inhibits the polymerization of actin monomers by binding in a 1:1 complex near the nucleotide-binding cleft [18], but it has also been shown to be highly ichthyotoxic [11, 16], antifungal [11] and cytotoxic against cancer cell lines [17]. A previous study demonstrated that, in addition to latrunculin A, laulimides (laulimalide and isolaulimalide) were also found in *Chromodoris lochi* [10], while puupehenone was been isolated from *C*. *elisabethina* [19]. Therefore, it would appear that the main chemical composition of the different species can differ, except for the co-occurrence of latrunculin A. This compound may play a critical role in the nudibranchs’ chemical defence mechanism and may be actively sequestered from dietary sponges and stored, while other compounds may be eliminated as part of the digestive process. However, little is known about the distribution of both latrunculin A and the other mentioned compounds in the different body parts of the nudibranchs.

Defensive compounds are often localized in peripheral body tissue of plants and animals, or at sites that are most frequently attacked by predators [20, 21]. At these locations, animals may sequester a suite of compounds that enhance the animal’s defense mechanism, whilst other species selectively uptake one or two compounds, whilst eliminating the others through the gut [20]. Selective sequestration has been studied in detail in insects and amphibians, e.g. [20, 22, 23]. Some nudibranch species have been shown to selectively bioaccumulate secondary metabolites from prey species [24, 25], and such storage of sequestered toxic metabolites for long periods is thought to be crucial to enable nudibranchs to compensate for prolonged separation from food [26]. Among the species that feed upon cnidarians, *Phyllodesmium guamensis* selectively sequesters the soft coral-derived cembranoid diterpene 11beta-acetoxypukalide within various tissue parts of its body, but actively excludes the related soft coral diterpene pukalide [27]. This may act as an alternative defensive strategy, based on the sequestration of bioactive chemicals from the prey rather than an accumulation of ‘cleptocnidae’ (cnidarians nematocysts), which is typical of aeolid nudibranchs [28, 29]. It has been also proved that opisthobranchs belonging to the family Chromodorididae are able to transfer selected metabolites from the food to their mantle tissues, storing them in defensive mantle dermal formations (MDFs) [30]. Other studies provided further evidence that sponge metabolites are selectively accumulated in the MDFs of chromodorid nudibranchs [21, 31], while other compounds, including lipids and steroids, were not detected in the MDFs, though they were found in different body parts of nudibranchs. Even in the absence of typical MDFs, defensive metabolites can be found accumulated in the exposed mantle rim of nudibranchs [32].

In this study, we investigated the anatomical distribution of latrunculin A in a range of closely-related [33] nudibranch species: *Chromodoris elisabethina*, *C*. *lochi*, *C*. *kuiteri*, *C*. *annae*, *C*. *magnifica*. Given the diffused and ramified structure of the MDFs variously localized along the mantle edge of the studied species, the whole mantle rim was dissected, and compared for its chemical composition with the rest of the mantle and the viscera (internal organs). We also conducted comparative toxicity, cytotoxicity, and feeding-deterrency assays with purified nudibranch compounds to examine the ecological significance of compounds found in each species.

## Materials and Methods

### Animal collection

**Fig 1:**
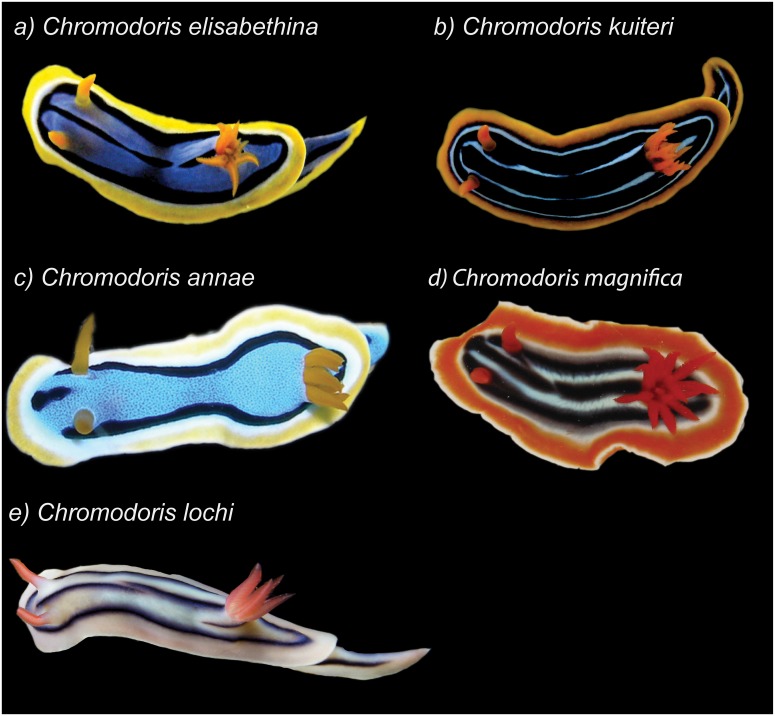
Photographs of study species displaying conspicuous colour patterns.

The following specimens were collected on SCUBA from reefs in Queensland (QLD), Australia: *Chromodoris elisabethina* (33 individuals, Mooloolaba, SE Queensland: 26°40’S; 153°07’E); *Chromodoris magnifica* (1 individual, Great Barrier Reef, exact location unknown, supplied by Cairns Marine Ltd, Stratford, QLD); *Chromodoris lochi* (2 individuals, Lizard Island, Great Barrier Reef: 14°40’S; 145°28’E; 1 individual from North Stradbroke Island, SE Queensland: 27°35’S; 153°27’E; 2 individuals from Mooloolaba); *Chromodoris kuiteri* (6 individuals, Mooloolaba); *Chromodoris annae* (3 individuals, Lizard Island) (Fig 1). Animals were collected under the following permits: Queensland General Fisheries Permit #161624; Great Barrier Reef Marine Park Authority G12/35688.1; Moreton Bay Marine Park Permit QS2012/MAN183. With the exception of *C*. *magnifica*, individuals were kept in aquaria for 24–48 hours after collection and then frozen and stored at −20°C. *C*. *magnifica* was kept for in aquaria for approx. 2 weeks.

### Dissection and extraction

Each nudibranch was carefully dissected into 2 or 3 parts: 1) viscera (internal organs); 2) mantle tissue; and in some individuals (*C*. *elisabethina*, *C*. *kuiteri* and *C*. *magnifica*), 3) the mantle rim, which incorporates MDFs [34], and is depicted by the yellow or orange border of nudibranchs in Fig 1a–1d, and the pink border of *Chromodoris lochi* in Fig 1e. The volume of each section was measured by the displacement of water in a graduated measuring cylinder or micro syringe, depending on size. The dissected tissues were separately chopped, extracted with acetone (3 ×10 mL) and sonicated (2 min). The extract was concentrated using a rotary evaporator before being partitioned between water (3 mL) and diethyl ether (4 × 4 mL) removing the organic layer. The extract was dried with anhydrous sodium sulfate, before concentration under nitrogen. Selected tissue extracts were subject to an anatomical quantification of compounds using methods adapted from [21]. The extracts were dissolved in deuterated chloroform for ^1^H NMR analysis on a Bruker AV-500 spectrometer. A capillary tube containing a known amount of 1,4-dimethoxybenzene was inserted into the NMR tubes as an internal standard. Integration of the signals at δ_H_ 6.84 resulting from the four aromatic protons of 1,4-dimethoxybenzene was used for quantification [21].

### Chemical identification and purification

**Fig 2:**
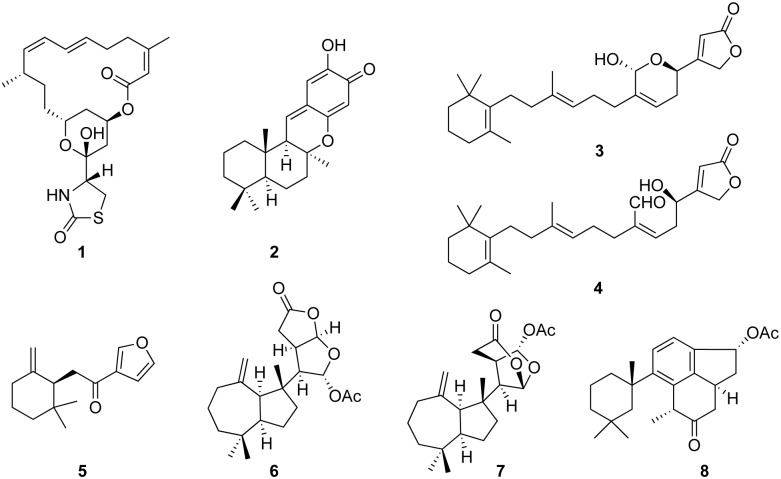
Structures of compounds identified. latrunculin A (**1**), puupehenone (**2**), deoxymanoalide (**3**) and deoxysecomanoalide (**4**), pallescensone (**5**), dendrillolide A (**6**), aplyviolene (**7**) and aplysulphurin (**8**).

Latrunculin A (**1**), puupehenone (**2**), pallescensone (**5**), dendrillolide A (**6**), and aplyviolene (**7**) (Fig 2) were identified from the ^1^H NMR and LRESIMS data of the crude extracts by comparison with the respective literature [6, 35–40]. Crude extracts containing deoxymanoalide (**3**) and deoxysecomanoalide (**4**) underwent normal phase flash column chromatography (Merck Silica gel 60) eluting from a gradient of hexanes/ethyl acetate to yield a semi-purified mixture. The mixture was further separated by normal phase high performance liquid chromatography (HPLC) using a Waters semi preparative column (μPorasil, 10 μm, 7.8×300 mm) eluting with 30:70 ethyl acetate/hexanes. Compounds **3**, **4** were then identified by a comparison of the ^1^H NMR data to the literature [41] in combination with LRESIMS measurements performed on a Bruker Esquire HCT instrument. The major metabolite found in the viscera of *C*. *annae* was provisionally identified as a linear furan related to ambliofuran [42], however the sample decomposed before its structural investigation could be completed.

For toxicity and unpalatability assays, all extracts containing latrunculin A were combined (60 mg) and subject to normal phase flash column chromatography (Merck Silica gel 60). Purification of latrunculin A was obtained by gradient elution using hexanes/ethyl acetate to yield 15 mg. The meroterpenoid puupehenone was obtained from the *Pseudoceratina* sp. 4909 (Verongida, Pseudoceratina) (identified by Dr. Merrick Ekins; QM accessions numbers: G319578, G319573, G335175). Sponge tissue (approximately 80 g) was finely chopped, extracted with acetone (3 x 50 mL) and sonicated (2 min). After concentration using a rotary evaporator the aqueous layer was partitioned with diethyl ether (4 × 15 mL), dried with sodium sulfate and concentrated under nitrogen to give 400 mg of extract. For comparison with other nudibranchs from the family Chromodorididae, but not found in this genus, we also purified the compound aplysulphurin (**8**), a major compound found in *Goniobranchus splendidus* [43]. Aplysulphurin was obtained from previous studies of *Goniobranchus reticulatus* [44, 45] and the sponge *Darwinella tango* [46]. All fractions containing aplysulphurin were combined (25 mg) to undergo HPLC (20:80 EtOAc/hexanes) resulting in 10 mg of the purified compound.

### Brine shrimp toxicity assay

We tested the relative toxic properties of purified compounds from each species of nudibranch with a brine shrimp toxicity assay, as per [47]. Brine shrimp eggs were hatched in a 500 mL beaker containing 400 mL artificial seawater (Tropic Marin), which was prepared using double distilled water, and kept under strong aeration and continuous illumination for approx. 30 hours. We prepared a stock concentration (approx. 12.0 mg/mL) of the compound with DCM. One glass microfiber filter paper (Whatman GF/C 47 mm diam.) was placed into individual glass petri dishes (55 mm diam.) then 0.005, 0.05, 0.5 mL of stock solution was transferred on to the filter papers with a glass pipette. The solvent was left to evaporate from the filter paper under a Nederman arm for 30 min. Twenty actively swimming instar I nauplii (< 12 h after hatching) were collected with a glass pipette and added to each petri dish with 5 mL filtered sea water. Lids were placed on top of the petri dishes and kept under constant illumination for 24 hours. Surviving nauplii (instar II/III) were then counted; nauplii were considered dead if no movement was detected after several seconds of observation. Natural mortality was controlled for using control treatments in which 0.5 mL of DCM was added to the filter paper. In all cases control deaths occurred, therefore the data was corrected using Abott’s formula % deaths = (test—control)/100 –control) for analysis [48].

### Cytotoxicity assay

The MTT (3-(4,5-dimethylthiazol-2-yl)-2,5-diphenyltetrazolium bromide) assay was modified from that previously described [49] using adherent NCIH460 (human large cell lung carcinoma), SW620 (human colorectal adenocarcinoma) and KB3-1 (human cervical carcinoma) cell lines. Briefly, cells were harvested with trypsin and dispensed into 96-well microtitre assay plates at 2,000 cells/well and incubated for 18 h at 37°C with 5% CO_2_ (to allow cells to attach). Compounds were dissolved in 5% DMSO in PBS (*v*/*v*) and aliquots (20 μL) were tested over a series of final concentrations ranging from 10 nM to 30 μM. Vinblastine was used as positive control and blank control wells were treated with 5% aqueous DMSO. After 68 h incubation at 37°C with 5% CO_2_, an aliquot (20 μL) of MTT in PBS (4 mg/mL) was added to each well (final concentration of 0.4 mg/mL), and the microtitre plates incubated for a further 4 h at 37°C with 5% CO_2_. After this final incubation the medium was aspirated and precipitated formazan crystals dissolved in DMSO (100 μL/well). The absorbance of each well was measured at OD_580 nm_ at r.t. on a POLARstar Omega microtitre plate reader. IC_50_ values were calculated using Prism 5.0 (GraphPad Software Inc., La Jolla, CA), as the concentration of analyte required for 50% inhibition of cancer cell growth (compared to negative controls). All experiments were performed in duplicate.

### Anti-feedant assay

Purified compounds found in the mantle and viscera of each nudibranch species were tested for their feeding deterrent activity against a common generalist shrimp species (*Palaemon serenus*). Shrimp were collected from intertidal zones in SE Queensland and housed communally in large aquaria until needed. Assays were conducted based on methods described in [21, 50, 51] using artificially dyed (red) food pellets treated with purified compounds of different concentrations. The pellets were made using mixture of ground freeze-dried squid mantle (50 mg), alginic acid (30 mg) and purified sea sand (30 mg). The pure compounds, dissolved in 0.5 mL of DCM, were added to the dry mixture and left for 30 min for the solvent to evaporate. One drop of red food colouring was added and 0.5 mL of distilled water. Food colouring was added for easy detection of the food in the digestive tract of the shrimp. The pellet ingredients were mixed carefully with a spatula, and then placed in to a 1mL syringe. The contents were extruded into a 0.25 M calcium chloride solution to create spaghetti-like strands and left for 2 min to harden. The pellets were then rinsed with distilled water and cut into pellets that were approx. 5 mm in length. Control foods were made in the same manner, with the addition of 0.5 mL of DCM, but without the purified metabolites.

Randomly selected shrimps (20–35 mm, total length) were individually placed in a section of an 8 compartment polypropylene translucent partitioned container (270 × 98 × 325 mm; each partition: 135 × 98 × 90 mm). Small holes were drilled in to the side of each container to allow water flow through the container to allow the shrimp to be housed for 1–2 weeks. Two partitioned boxes were placed in larger aquaria (650 × 350 × 350 mm) with 15 cm depth of seawater and air stones to create water circulation. Shrimp were left for 3 days to acclimatize and fed green fish flakes (Ocean Nutrition, Formula 2) once per day. Shrimp were then starved for 3 days before assays began. Ten randomly selected shrimp were given a coloured pellet of a particular concentration with a pair of tweezers. Most shrimp readily accepted the pellet with it their clawed appendages (chelipeds). The shrimps were then monitored at 5, 10, 30, 60 min after being given the pellet, and we recorded whether the shrimp appeared to be eating the pellet, or not eating. After 60 min, the presence of a red spot in the transparent gastric mill was considered acceptance of the pellet by the shrimp, while the absence of a red spot was considered a rejection. If a shrimp had rejected the pellet, it was then given a control pellet and again observed for 30 min. If the shrimp rejected the control pellet (< 1%), then that shrimp was removed from the final analysis, as these shrimp were often in the process of molting or about to expire. Shrimp were not reused. Tanks were cleaned and water replaced before the next assay commenced. The assay was repeated for each compound 3–4 times, and the mean % acceptance rate was calculated. Again, we calculated the effective dose response curve and calculated the concentration at which 50% of the pellets were rejected by the shrimp (ED_50_).

## Results

### Anatomical distribution of compounds

The only metabolite found within both the mantle and mantle rim of every nudibranch species in this study was the macrolide latrunculin A (**1**) (Table 1). Compared to concentrations in the viscera, latrunculin A was more concentrated in the mantle, and reached its highest concentration in the mantle rim (Table 2). In the viscera, *C*. *elisabethina* specimens contained only the metabolite puupehenone (**2**), while *C*. *magnifica* contained both **2** and minor amounts of **1**. *C*. *lochi* specimens contained manoalide compounds with deoxymanoalide (**3**) and deoxysecomanoalide (**4**) in the viscera. *C*. *kuiteri* contained the sesquiterpene pallescensone (**5**) and the diterpenes dendrillolide A (**6**) and aplyviolene (**7**) (Table 1; Fig 2). *C*. *annae* contained an unidentified linear furan as the main metabolite (Table 1). ^1^H NMR spectra for crude extracts of each species (except *C*. *annae*) are shown in S1–S4 Figs, which contain 1,4-dimethoxybenzene (DMB) as an internal standard.

**Table 1:** Anatomical distribution of compounds from *Chromodorididae* nudibranchs.

Species	Compound		
	Mantle rim	Mantle	Viscera (including digestive system)
***C*. *elisabethina***	**1**	**1**	**1, 2**
***C*. *magnifica***	**1**	**1**	**1, 2**
***C*. *lochi***	^‡^	**1**	**3, 4**
***C*. *kuiteri***	**1**	**1**	**5, 6, 7**
***C*. *annae***	^‡^	**1**	*

^‡^Rim was not dissected from mantle in these species

*unidentified metabolites

**Table 2:** Quantification of the metabolites within the each tissue. Values shown are mean ± s.e.; number of individuals in parentheses.

Species	Compound	Mantle rim (mg/mL)	Mantle (mg/mL)	Viscera (mg/mL)
***C*. *elisabethina* (n = 3)**	1	8.7 ± 2.7	2.1 ± 1.1	0.2 ± 0.2
	2	-	-	1.2 ± 0.4
***C*. *magnifica* (n = 1)**	1	45.1	7.8	0.2
	2	-	-	0.2
***C*. *lochi* (n = 2)**	1	1.7 ± 1.0	1.2 ± 0.8	-
	3	-	-	90.5 ± 22.7
	4	-	-	7.0 ± 2.3
***C*. *kuiteri* (n = 1)**	1	26.2	12.2	-
	5	-	-	2.7
	6	-	-	1.7
	7	-	-	0.7

### Brine shrimp toxicity assay

**Fig 3:**
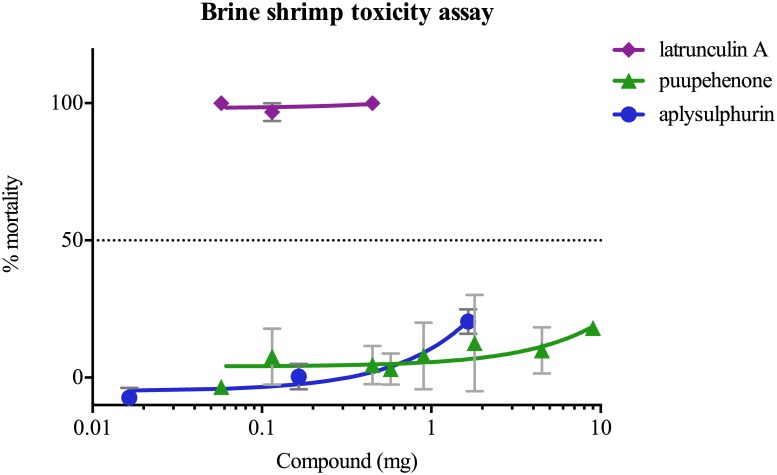
Brine shrimp toxicity assay. mortality of brine shrimp (%) with increasing concentrations of compound.

Latrunculin A was significantly more toxic to brine shrimp than puupehenone and aplysulphurin (Fig 3). Even relatively small amounts of latrunculin A (0.06 mg per treatment) caused 100% mortality of brine shrimp, which were much lower concentrations than that found in the rim of the nudibranchs (Table 2). There was no significant response in mortality of brine shrimp from puupehenone or aplysulphurin, even at concentrations of up to 9.0 mg per treatment (puupehenone) or 1.6 mg (aplysulphurin).

### Cytotoxicity assay

**Fig 4:**
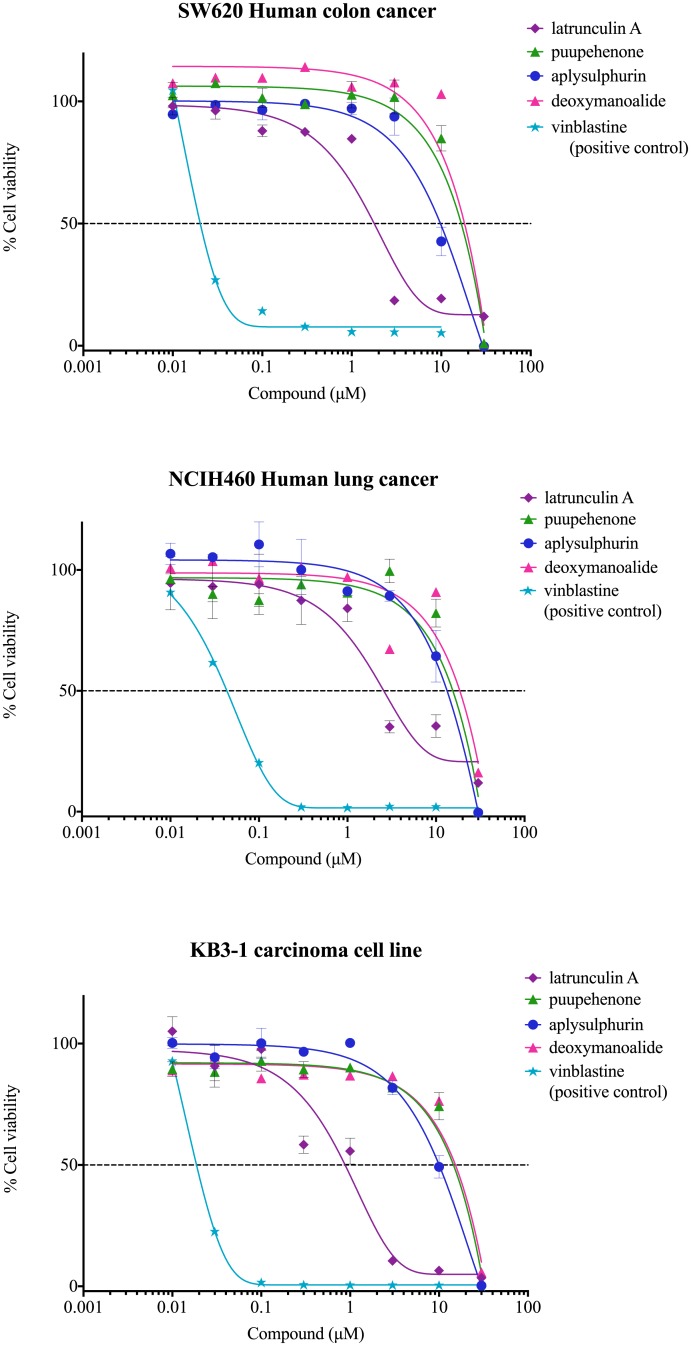
Cytotoxic activities. against SW620, NCHI420 and KN3-1 cancer cell lines.

Latrunculin A was the most potent of the isolated nudibranch compounds used against all cell lines, with IC_50_ values of 1.9, 2.7, and 0.9 μM for cell lines SW620, NCIH460 and KB3-1, respectively. Aplysulphurin was the second most potent, with IC_50_ values of 10.5, 13.7, 9.8 μM. Although puupehenone has previously shown significant cytotoxic activity against A-549 and HT-29 neoplastic cell lines [52], in this study there was little effect with IC_50_ values of > 16.5 μM. Deoxymanoalide also had high IC_50_ values of > 15.7 μM (Fig 4).

### Anti-feedant assays

**Fig 5:**
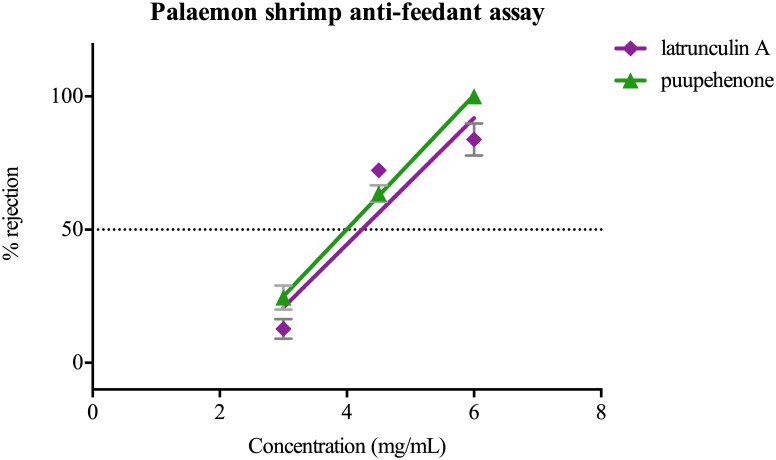
Anti-feedant assay. rejection of pellets (%) by palaemon shrimp, *Palaemon serenus*.

Latrunculin A and puupehenone produced a similar feeding deterrent response against the shrimp, *Palaemon serenus*. ED_50_ values for latrunculin A were 4.2 mg/ml and 4.0 mg/ml for puupehenone within food pellets (Fig 5). The deterrent response against latrunculin A occurred at concentrations much lower than what was found in the mantle rim of *C*. *elisabethina*, *C*. *kuiteri* and *C*. *lochi* (Table 2).

## Discussion

In this study, we show that a group of closely related nudibranch species, belonging to the genus *Chromodoris*, sequester and store the unpalatable and toxic compound latrunculin A. This compound was found at high concentrations in the rim of the mantle, where MDFs are situated [34], and is therefore at the first line of attack by potential predators [21]. Surprisingly, latrunculin A was only found in very small amounts in the viscera, and only in a few individuals. Instead, a variety of other compounds were found in the viscera of these species, including: puupehenone in *C*. *elisabethina* and *C*. *magnifica*; manoalides in *C*. *lochi*; a linear furan in *C*. *annae*; pallescensone and dehydroherbadysidolide in *C*. *kuiteri*. This suggests that sponges containing latrunculin A are not the main dietary food source of this group of nudibranchs (at least at our collection sites), and implies less specialized alimentary habits than previously thought [14]. Indeed, a previous study indicated that *C*. *elisabethina* fed on a brown encrusting sponge, *Heteronema* sp., which contained puupehenone [11] but did not show any traces of latrunculin A. *C*. *elisabethina* was also shown to feed on *Dysidea* sp. in the Marshall Islands [53], which also do not contain latrunculin A to the best of our knowledge. Such a selective sequestration mechanism seems to allow these nudibranchs to feed on multiple prey sponges, whilst still obtaining an optimal and stable chemical profile for defensive purposes.

How such a selective sequestration mechanism evolves is intriguing. These animals obtain their food and means of defending themselves by eating organisms that are toxic. Although it has been hypothesized that dietary toxic compounds were primarily compartmentalized by mollusks to circumvent autotoxicity (i.e. self-intoxication) [34, 54], Faulkner & Ghiselin proposed that at first the harmful sponge metabolites were probably excreted [55], while a variety of dorsal glands evolved later with the development of mechanisms for storage and effective delivery of harmful sponge metabolites. We propose that, in order for animals to feed on toxic sponges, these animals may have developed specific detoxification mechanisms that enable them to transport toxic compounds in the hemolymph to be excreted. Some reversible mechanism, possibly by conjugation with polar ligands, could have facilitated the transport of the compounds by increasing their solubility, as proposed for the elimination of xenobiotics in fish species [56]. The more ancestral species might have then become capable of reactivating the chemical weapon upon its secretion outside the body, and this may have preceded the bioaccumulation phenomena in mantle dermal formations seen in more derived species [34]. The storage of chemical weapons in confined parts of the body may have therefore evolved later as a defensive function, allowing the evolutionary optimization of resource allocation, with the accumulation of progressively increasing concentrations of distasteful metabolites in exposed sacrificial body parts [31]. This hypothesis is supported by the fact that the nudibranchs are able to handle toxic compounds without suffering adverse effects and distribute them in their secretions, sacrificial parts of the body, and in egg masses masses [25, 57].

Latrunculin A was more toxic both *in vitro* and *in vivo* than other compounds isolated from nudibranchs in this study and from a compound (aplysulphurin) isolated from other Chromorodoridiae nudibranchs such as *Goniobranchus splendidus* [43] and *Goniobranchus reticulatus* [44, 45]. It would therefore be a prime target for ‘deactivation’ before damage to the animal occurs. The mechanism for both detoxification and transport of this foreign compound is, however, unknown and deserves further investigation. The toxicity of latrunculin A to both brine shrimps and cancer cell lines is consistent with the compound affecting cellular metabolism, being most effective at killing cells that are rapidly dividing, as a result of disruption of actin polymerization [18, 58]. However, in addition to the toxicological and pharmacological interest, our findings suggest that the digestive system of the studied nudibranchs is especially adapted to detoxify this compound. One reason why certain nudibranchs can eat only a few types of sponges could depend, in fact, on their ability to cope with different toxins.

In feeding assays, latrunculin A was equally as unpalatable as the compound puupehenone, which was found in the viscera of two of the studied species. During anti-feedant assays, shrimps appeared to test the artificial food with their chemosensory organs (antennules) before deciding whether to accept or reject it. This suggests that latrunculin A can, through taste or olfaction, act as reliable chemosensory aposematic signal as it associated with toxicity. In 1980, Eisner and Grant suggested that odors could act as warning signals and this was termed olfactory aposematism [59]; however, at that time they did not find evidence that odours themselves were intrinsically repellent to predators or played a direct role in chemical defense. However, it has been recently proposed that chemical secretions can act both as a signal, and as a secondary defence component [60, 61]. Although the term olfaction becomes ambiguous when referred to aquatic life, chemosensory perceptions of lipophilic compounds, some of which are naturally odorant, are known to occur among marine organisms [62]. The low-solubility of these compounds in water however enhances efficacy of short-range communication in water by limiting the dilution of the signal in the medium. Chemosensory cues may be more important than visual signals when protecting prey individuals from predators with poor visual systems that are unlikely to detect conspicuous colour patterns.

Each of our study species display highly conspicuous colour patterns (Fig 1) [63], which are thought to act as visual warning signals to potential predators. These species could be part of a visual Müllerian mimetic ring of closely related individuals, in which similar colour patterns strengthens predator avoidance learning. Analogously, the existence of visual Müllerian mimicry has already been proposed for a group of several blue, white, and yellow Mediterranean and northeastern Atlantic species of chromodorids, and supported by chemoecological studies which revealed the involvement of a variety of defensive compounds, often characterizing individual species [32]. However, here we also show that the occurrence of the same defensive chemosensory warning signal could describe, for the first time, a case of chemosensory Müllerian mimicry within marine organisms. In contrast to the Mediterranean and northeastern Atlantic group of nudibranchs, the presence of latrunculin A seems to be a common feature of the species examined in the current study, strongly suggestive of the occurrence of both a visual and chemosensory Müllerian mimicry.

Although this study demonstrates that conspicuous displays indicate a strong level of chemical defence, the evolutionary question as to whether different aposematic signals within groups are ‘quantitatively honest’, in the sense that conspicuousness correlates positively with levels of toxicity and thus conveys reliable information [64] requires further investigation. A more extensive comparative study would be needed to examine the shape of the relationship between signal strength and toxicity to assess the implications for ecological processes and evolution.

## Conclusion

A group of conspicuously colored spongivorous nudibranchs belonging to the genus *Chromodoris* accumulate the dietary 16-membered macrolide latrunculin A for defensive purposes, a combination of biological assays showing that the compound act both as a feeding deterrent and as a toxic chemical weapon. This implies that a group of chromodorid nudibranchs form a putative Müllerian mimetic circle, based on both a common chemosensory signal, latrunculin A, and similar conspicuous visual signals. Although it is widely accepted that Müllerian mimicry need not to encompass visual mimicry only, but may employ any of the senses, our work, for the first time, highlights the existence of a group of aquatic organisms that use the same aposematic chemosensory signal. Latrunculin A evidently represents at the same time an aposematic signal and, given its high toxicity, it determines the unprofitability of the studied nudibranch species. This signal could be detected by a wide variety of potential predators, including those devoid of well-developed visual systems, therefore unable to detect the color patterns of the nudibranchs.

## Supporting Information

S1 Fig ^1^H NMR spectra (500 MHz, CDCl_3_) of *C*. *kuiteri* (#1280) crude extract. (DOCX)

S2 Fig ^1^H NMR spectra (500 MHz, CDCl_3_) of *C*. *lochi* (#1272) crude extract. (DOCX)

S3 Fig ^1^H NMR spectra (500 MHz, CDCl_3_) of *C*. *magnifica* (#432) crude extract. (DOCX)

S4 Fig ^1^H NMR spectra (500 MHz, CDCl_3_) of *C*. *elisabethina* (#552) crude extract. (DOCX)
