# Supplementary material for: Choose Your Weaponry: Selective Storage of a Single Toxic Compound, Latrunculin A, by Closely Related Nudibranch Molluscs
Source: PLoS One. 2016 Jan 20;11(1):e0145134. doi: 10.1371/journal.pone.0145134 (PMC4720420; doi:10.1371/journal.pone.0145134)
Supplement: S1 Fig — (DOCX) [file pone.0145134.s001.docx]

**S1 Fig**. ^1^H NMR spectra (500 MHz, CDCl_3_) of *C. kuiteri* (#1280) crude extracts containing 1,4-dimethoxybenzene (DMB) as an internal standard.


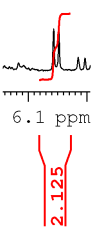

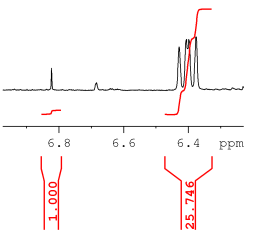

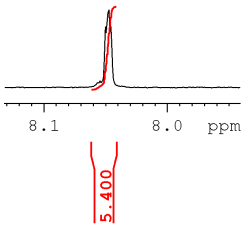

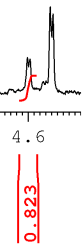

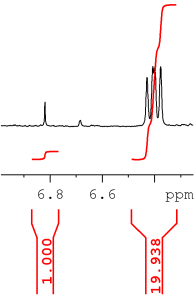

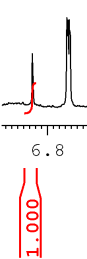

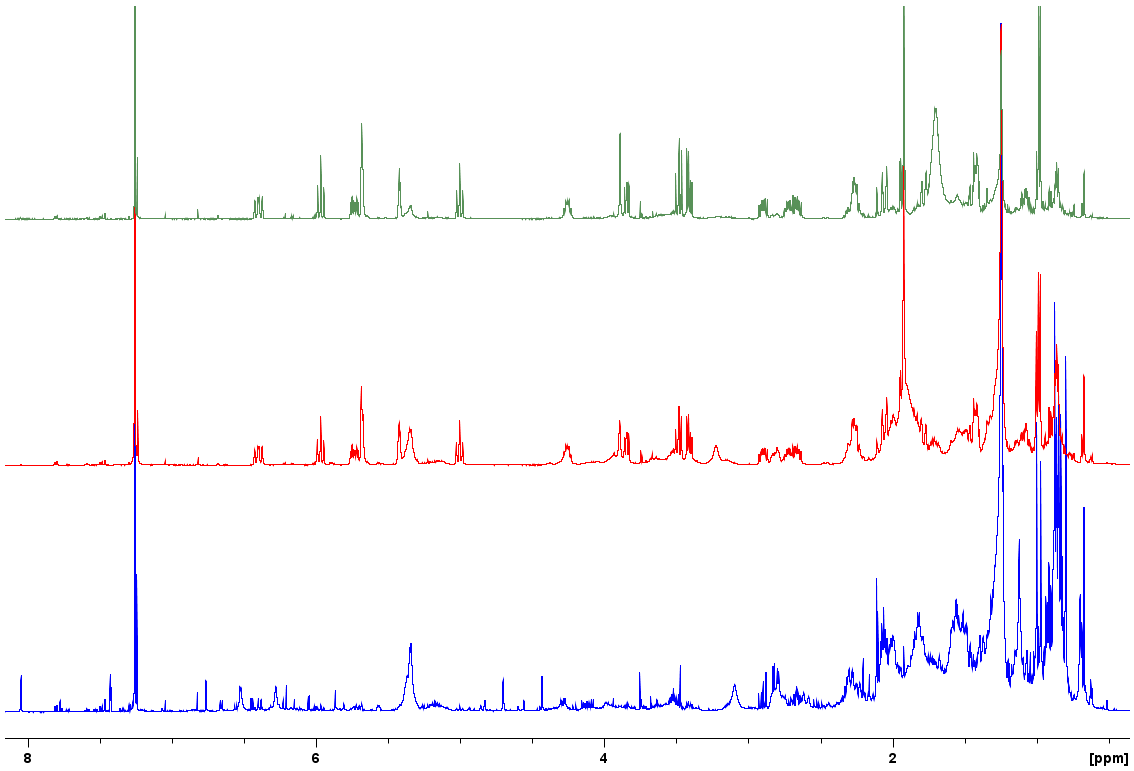


**H20’’’**

**H16’’**

**H7**

**DMB**

**H2’**

**DMB**

**Viscera**

**Mantle**

**Mantle rim**
